# Overcoming delivery barriers of large all-in-one CRISPR/Cas9 plasmids using lysine-based lipid nanoparticles

**DOI:** 10.3389/fgeed.2026.1888079

**Published:** 2026-09-16

**Authors:** Wenhan Sung, Tianshu Li, Shinji Takeoka

**Affiliations:** 1 Department of Life Science and Medical Bioscience, Graduate School of Advanced Science and Engineering, Waseda University, Tokyo, Japan; 2 Liberal Arts and Sciences Education Center, Musashi University, Tokyo, Japan; 3 Institute for Advanced Research of Biosystem Dynamics, Research Institute for Science and Engineering, Waseda University, Tokyo, Japan

**Keywords:** aminolipids, biocompatibility, cationic lipids, CRISPR/Cas9, lipid nanoparticles (LNPs), non-viral gene delivery

## Abstract

**Introduction:**

Lipid nanoparticles (LNPs) are widely used as drug delivery systems (DDS) for the intracellular delivery of gene-editing tools such as the clustered regularly interspaced short palindromic repeats (CRISPR)/CRISPR-associated protein 9 (Cas9) system. Previously, a lysine-headgroup cationic lipid (K3C16) was reported to exhibit high biocompatibility and cellular internalization via plasma membrane fusion when formulated as liposomes, suggesting its potential as a low-toxicity gene delivery material. However, the translation of this lipid into a multi-component lipid nanoparticle (LNP) matrix via microfluidic engineering for large cargo encapsulation has not been explored. In this study, K3C16 was successfully engineered for the first time as the main lipid component of LNPs to deliver large plasmid DNA encoding the CRISPR/Cas9 system.

**Methods:**

First, 1,2-dioleoyl-sn-glycero-3-phosphoethanolamine (DOPE) and 1,2-dioleoyl-sn-glycero-3-phosphocholine (DOPC) were evaluated as helper phospholipids to identify an optimal lipid composition. LNPs containing DOPE exhibited significantly higher transfection efficiency than those containing DOPC and were therefore selected for subsequent experiments. Next, LNPs formulated with different cationic or ionizable lipids were prepared and evaluated in HEK293 cells for transfection efficiency, cytotoxicity, and gene-editing efficiency.

**Results:**

LNPs containing the commercial ionizable lipid SM-102 showed higher transfection and gene-editing efficiencies than the lysine-headgroup cationic lipids represent a promising and biocompatible platform for CRISPR/Cas9 plasmid delivery; however, increased cytotoxicity was observed in highly transfected cell populations. In contrast, the lysine-headgroup cationic LMPs achieved effective delivery of large plasmid DNA and CRISPR/Cas9-mediated gene editing while maintaining superior biocompatibility.

**Conclusion:**

These results demonstrate that lysine-headgroup cationic lipids represent a promising and biocompatible platform for CRISPR/Cas9 plasmid delivery. Further optimization of lipid composition and molar ratios may enhance transfection and gene-editing efficiencies, advancing the development of safer nonviral gene delivery systems.

## 1 Introduction

The clustered regularly interspaced short palindromic repeats (CRISPR)/CRISPR-associated protein 9 (Cas9) system has emerged as a powerful and versatile genome-editing tool (Lander, 2016), representing the third generation of gene-editing technologies following zinc-finger nucleases (ZFNs) (Miller et al., 2007) and transcription activator-like effector nucleases (TALENs) (Christian et al., 2010). Originally discovered as a part of the adaptive immune system in bacteria and archaea, the CRISPR/Cas9 mechanism enables these organisms to defend against invading nucleic acids (Horvath and Barrangou, 2010). The system consists of the Cas9 nuclease and a single guide RNA (sgRNA), which directs Cas9 to a target DNA sequence adjacent to a protospacer-adjacent motif (PAM). Upon binding, Cas9 introduces a site-specific double-strand break (DSB) that is subsequently repaired by the host cell through either non-homologous end joining (NHEJ) or homology-directed repair (HDR) (Hsu et al., 2014). This programmable and efficient editing capability makes CRISPR/Cas9 a promising platform not only for disease modeling (Matano et al., 2015) but also for therapeutic applications in clinical medicine (Dever et al., 2016). In the early stages of CRISPR/Cas9 development, delivery strategies primarily relied on physical approaches such as microinjection and electroporation, as well as viral vectors including lentivirus and adeno-associated virus (AAV) (Li et al., 2018). While these methods can achieve efficient delivery, physical approaches are limited to *in vitro* use and often cause substantial cellular damage (Chen et al., 2016), whereas viral vectors, despite ensuring stable transgene expression, pose safety concerns such as insertional mutagenesis, immune activation, and high production complexity (Sun et al., 2003; Li et al., 2015). Consequently, non-viral delivery systems have attracted increasing attention owing to their superior biocompatibility, lower immunogenicity, and scalable manufacturing potential. Among them, lipid-, polymer-, and peptide-based carriers have emerged as the most widely studied platforms, capable of improving physicochemical stability, cellular uptake, and intracellular release of CRISPR/Cas9 components (Rouatbi et al., 2022).

Lipid nanoparticles (LNPs) have emerged as a promising drug delivery system (DDS) for nucleic acid-based therapeutics such as small interfering RNA (siRNA), plasmid DNA (pDNA), and messenger RNA (mRNA) (Liebertpub, 2026). Among these, mRNA-based LNP vaccines have achieved remarkable success during the COVID-19 pandemic, exemplified by those developed by Pfizer-BioNTech (Polack et al., 2020) and Moderna (Baden et al., 2021). The key component of these formulations is the ionizable lipid, which plays a critical role in mediating nucleic acid encapsulation and intracellular delivery. Ionizable lipids acquire a positive charge under acidic conditions during formulation, allowing electrostatic complexation with negatively charged nucleic acids. At physiological pH, they become nearly neutral, which minimizes nonspecific interactions with serum proteins and prevents protein-induced aggregation. Upon endocytosis, the acidic endosomal environment re-protonates the ionizable lipid, promoting electrostatic interaction with the endosomal membrane and facilitating endosomal escape of the encapsulated nucleic acids (Schlich et al., 2021).

Currently, most LNP formulations are optimized for mRNA delivery rather than for pDNA. While mRNA delivery typically induces transient protein expression (Moseman et al., 2025), pDNA delivery offers prolonged gene expression and even genomic integration (Davis, 1997), features particularly advantageous for sustained CRISPR/Cas9-mediated genome editing. A significant advancement in non-viral CRISPR delivery is the utilization of “all-in-one” plasmid constructs. Unlike multi-component strategies that require the co-delivery of separate Cas9 mRNA and sgRNA species, all-in-one vectors consolidate the Cas9 nuclease, the sgRNA expression cassette, and often a fluorescent reporter into a single genetic backbone. This architecture ensures stoichiometric consistency within every transfected cell (Ran et al., 2013; Yin et al., 2017a), bypassing the complexities of varied encapsulation efficiencies and differing degradation rates often seen in co-delivery systems.

In our laboratory, a series of amino acid–based cationic lipids have been developed, each consisting of a hydrophilic amino acid-derived headgroup, a spacer, and two hydrophobic tails linked by glutamic acid (Obata et al., 2009). Among them, the lysine-type cationic lipids; K3C14 and K3C16 (Figure 1), exhibit low cytotoxicity when prepared into liposomes, while maintaining a positive charge at a physiological pH by a high p*K*_*a*_ of the primary amine group within the lysine headgroup ( α -amine: p*K*_*a*_ = 8.95, ϵ -amine: p*K*_*a*_ = 10.53) (Nelson and Cox, 2025). Notably, K3C16 assemblies have been reported to promote membrane fusion, suggesting an alternative cellular entry route distinct from classical endocytosis (He et al., 2019). Based on these properties, K3C16 represents a promising candidate for the delivery of large pDNA constructs encoding the CRISPR/Cas9 system, potentially enabling enhanced nucleic acid loading through higher surface charge and more efficient delivery via membrane fusion. To date, non-viral delivery engineering has focused almost exclusively on ionizable lipid matrices optimized for short mRNA or RNP formats, leaving the unique biophysical packaging constraints of rigid, all-in-one plasmid DNA unaddressed. Here, we demonstrate that lysine-headgroup cationic LNPs serve as an effective and safe delivery platform specifically tailored for large CRISPR/Cas9 plasmid vectors.

**FIGURE 1:**
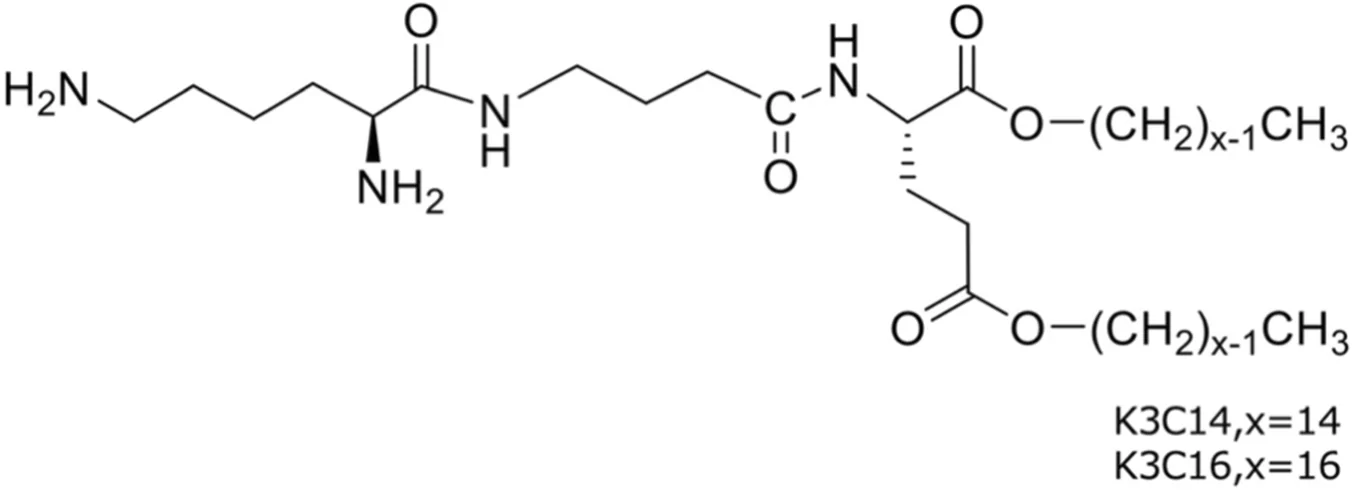
Chemical structures of synthesized lysine-headgroup cationic lipids. Both molecules share a same hydrophilic lysine headgroup, a hydrocarbon spacer, and a glutamic acid backbone linker, but differ in the length of their dual hydrophobic tails: K3C14 features 14-carbon alkyl chains, whereas K3C16 features longer 16-carbon alkyl chains.

In this study, we aimed to evaluate the gene-editing capability of lysine-based lipid nanoparticles (LNPs) for the delivery of CRISPR/Cas9 plasmid DNA. LNPs were first formulated using a microfluidic device with a lysine-headgroup cationic lipid (K3C16) as the primary cationic lipid and were screened using two different helper phospholipids (DOPC or DOPE) to identify an optimal lipid composition. After selecting the helper phospholipid, additional LNP formulations were prepared by incorporating a lysine-headgroup cationic lipid (K3C14 or K3C16), or a commercial cationic lipid (DOTAP), or a commercial ionizable lipid (SM-102), using the pGuide-it plasmid encoding Cas9, guide RNA, and the fluorescent reporter ZsGreen. The resulting formulations were evaluated in HEK293 cells, and their transfection efficiency, cytotoxicity, and CRISPR/Cas9-mediated gene-editing efficiency were comprehensively assessed.

## 2 Materials and methods

### 2.1 Materials

K3C14 and K3C16 were synthesized in our laboratory. 1,2-Dimyristoyl-rac-glycero-3-methoxypolyethylene glycol-2000 (PEG2000-DMG) was purchased from Avanti Research™ (Alabama, United States). 1,2-dioleoyl-sn-glycero-3-phosphoethanolamine (DOPE), 1,2-dioleoyl-sn-glycero-3-phosphocholine (DOPC) and Cholesterol were purchased from Tokyo Chemical Industry Co., Ltd. (Tokyo, Japan). 1,2-Dioleoyl-3-trimethylammonium-propane (DOTAP) was purchased from. Heptadecan-9-yl 8-((2-hydroxyethyl)(6-oxo-6-(undecyloxy)hexyl)amino)octanoate (SM-102) was purchased from Echelon Biosciences, Inc. (Massachusetts, United States). Ethanol was purchased from Fujifilm Wako Chemicals (Osaka, Japan). Plasmid DNA pCAG-EGFP (#89684) and pCas9-GFP (#44719) were purchased from Addgene (United States). Guide-it™ CRISPR/Cas9 System (Green), NucleoBond® Xtra Midi, and lysis buffer were purchased from Takara Bio Inc. (Shiga, Japan). The content of Guide-it™ CRISPR/Cas9 System (Green) included pGuide-it-ZsGreen1 Vector (Linear), DNA Ligation Mighty Mix, Guide-it Oligo Annealing Buffer, Guide-it Sequencing Primer q, PCR-Grade Water, Stellar Competent Cells (100 µL/tube), and SOC Medium. The gRNA sequence (Target:CCR5, Target sequence: 5′-AGT​TTA​CAC​CCG​ATC​CAC​TG-3′, PAM: 5′-GGG-3′)) was designed by CHOPCHOP (Labun et al., 2019), and the Oligo DNA (sense:5′-ccggAGTTTACACCCGATCCACTG-3′, antisense:5′-aaacCAGTGGATCGGGTGTAAACT-3′) was synthesized by Funakoshi Co., Ltd. (Tokyo, Japan). Primers for PCR (forward: 5′-TCG​GGG​AGA​AGT​TCA​GAA​ACT​A-3′ reverse: 5′-CCC​AGG​CTG​TGT​ATG​AAA​ACT​A-3′) were synthesized by Eurofins Genomics Japan (Tokyo, Japan). 2-[4-(2-Hydroxyethyl)-1-piperazinyl]ethanesulfonic acid (HEPES) was purchased from Dojindo Laboratories (Kumamoto, Japan). Amicon® Ultra-15 Centrifugal Filter Units were purchased from Merck Millipore (Massachusetts, United States). HEK293 cell was purchased from JCRB cell bank (Osaka, Japan). Dulbecco’s Modified Eagle Medium (DMEM), Dulbecco’s phosphate-buffered saline (DPBS), penicillin-streptomycin (PS), Lipofectamine® 3000 Reagent, and Quant-iT™ PicoGreen™ dsDNA reagent were purchased from ThermoFisher Scientific (Massachusetts, United States). Fetal Bovine Serum (FBS) was purchased from Sigma-Aldrich (Massachusetts, United States). 7-Amino-Actinomycin D (7-AAD) was purchased from BD Bioscience (New Jersey,United States). 2X PCR Master Mix was purchased from New England Biolabs (Massachusetts, United States). QIAquick PCR Purification Kit was purchased from QIAGEN N.V. (Venlo, Netherlands).

### 2.2 Construction of Cas9 plasmid DNA

#### 2.2.1 Annealing oligos

The C-C chemokine receptor type 5 (CCR5) gene was selected as our target sequence because it serves as the primary co-receptor required for HIV-1 viral entry and stands as a well-established, clinically relevant benchmark target for validating the disruptive knockout efficiency of novel non-viral CRISPR/Cas9 delivery platforms. Sense and antisense gRNA oligonucleotides were resuspended in nuclease-free water to a final concentration of 100 µM. For annealing, 1 µL of each oligo solution was combined with 8 µL of Guide-it™ Oligo Annealing Buffer in a 200 µL PCR tube. The mixture was subjected to thermal annealing using an Applied Biosystems Veriti™ 96-Well Thermal Cycler (Massachusetts, United States) under the following conditions: 95 °C for 2 min, followed by a gradual cooling ramp from 85 °C to 30 °C over 10 min, and then held at 25 °C. After annealing, 1 µL of the annealed oligo mixture was diluted with 99 µL of Guide-it™ Oligo Annealing Buffer to obtain a 100 nM (fmol/µL) solution. The annealed gRNA was stored at −20 °C until further use.

#### 2.2.2 Cloning the sgRNA targeting sequence into the pGuide-it vector

Annealed oligonucleotides (1 µL) were mixed with 2 µL of pGuide-it™ Vector (Linear), 2 µL of PCR-grade water, and 5 µL of DNA Ligation Mighty Mix. The ligation reaction was incubated at 16 °C for 30 min. Meanwhile, one vial of Stellar™ Competent Cells was thawed on ice. Following ligation, 10 µL of the reaction mixture was gently added to the competent cells and mixed by tapping, then incubated on ice for 30 min. The cells were heat-shocked at 42 °C for 45 s, followed by 2 min of incubation on ice. After recovery, the cells were plated on pre-warmed (37 °C) LB agar plates containing ampicillin (100 μg/mL) and incubated overnight at 37 °C. After incubation, 2–3 single colonies were picked and inoculated into LB medium containing ampicillin (100 μg/mL), followed by shaking incubation overnight at 37 °C using a TAITEK BIO-SHAKER BR-40LF (Saitama, Japan). Plasmid DNA was extracted using the NucleoBond® Xtra Midi Plasmid DNA Purification Kit according to the manufacturer’s protocol. The plasmid concentration was quantified using a Shimadzu BioSpec-nano spectrophotometer (Kyoto, Japan), and sequence verification was performed by Sanger sequencing using the Guide-it™ Sequencing Primer 1.

### 2.3 Preparation of lipid nanoparticles

Mixed lipids (Table 1) were dissolved in ethanol to a total lipid concentration of 9 mM at a molar ratio of 50:10:38.5:1.5. The lipid mixture was prepared in a total volume of 200 μL. Plasmid DNA (pCAG-EGFP, pCas9-GFP, or pGuide-it) was dissolved in 600 μL of buffer at a concentration corresponding to an N/P (nitrogen-to-phosphate) ratio of 5. For formulations containing cationic lipids, pDNA was dissolved in 20 mM HEPES buffer (pH 7.4), whereas for ionizable lipid formulations, pDNA was dissolved in 25 mM sodium acetate buffer (pH 4.0). The ethanolic lipid solution and the aqueous pDNA solution were mixed using a Darwin staggered herringbone micromixer (Paris, France) at a total flow rate of 3.6 mL/min with a buffer-to-ethanol flow rate ratio of 3:1. Following microfluidic mixing, the resulting LNP dispersion was diluted with pH 7.4 HEPES buffer and concentrated using Merck Amicon® Ultra-15 centrifugal filters (30 kDa MWCO, Darmstadt, Germany) to reduce the residual ethanol content to below 1%.

**TABLE 1:** Composition of lipid nanoparticles.

LNP sample	pDNA	Lipid composition (molar ratio = 50/10/38.5/1.5)
K3C16-DOPC LNP (pCas9)	pCas9-GFP (9270 bp)	K3C16/DOPC/Chol/PEG-DMG
K3C16-DOPE LNP (pCas9)		K3C16/DOPE/Chol/PEG-DMG
K3C16-DOPE LNP (pCAG)	pCAG-EGFP (5521 bp)	K3C16/DOPE/Chol/PEG-DMG
K3C14 LNP	pGuide-it (8327 bp)	K3C14/DOPE/Chol/PEG-DMG
K3C16 LNP		K3C16/DOPE/Chol/PEG-DMG
DOTAP LNP		DOTAP/DOPE/Chol/PEG-DMG
SM-102 LNP		SM-102/DOPE/Chol/PEG-DMG

### 2.4 Characterization of lipid nanoparticles

Particle sizes, polydispersity index (PDI), and zeta potential were measured using Malvern Zetasizer NanoZS90 or Malvern Zetasizer Pro (Worcestershire, England) after diluting the samples 500-fold with HEPES buffer (pH7.4). pDNA loading efficiency was quantified using Invitrogen Quant-iT™ PicoGreen™ dsDNA Assay Kit (Massachusetts, United States). For standard calibration, pDNA standards were prepared according to the manufacturer’s instructions. For LNP samples, a 10-fold diluted LNP dispersion was used. Briefly, 50 μL of each pDNA standard or diluted LNP sample was transferred into a 1.5 mL microtube. To disrupt the LNP structure and release the loaded pDNA, 50 μL of 2% Triton X-100 solution was added. For comparison, an equivalent volume of Tris–EDTA (TE) buffer was added to parallel samples to quantify free pDNA. After vortex mixing, samples were incubated at 40 °C for 15 min. Subsequently, 900 μL of PicoGreen reagent diluted 200-fold in TE buffer was added to each sample. The mixtures were then transferred into a black 96-well plate with a final volume of 200 μL per well, and each sample was measured in triplicate. Fluorescence intensity was recorded using a BioTek Instruments Synergy H1 microplate reader (Vermont, United States) with an excitation wavelength of 480 nm and an emission wavelength of 520 nm. The pDNA concentration was calculated based on the standard calibration curve. Loading efficiency was determined by comparing the total pDNA concentration measured after Triton X-100 treatment with the pDNA concentration measured in samples treated with TE buffer (Equation 1).

$$\text{Loading efficiency }\left(\%\right)=\frac{\text{TX}100\;\text{treated}\;\text{LNP}-\text{TE}\;\text{treated}\;\text{LNP}\;}{\text{TX}100\;\text{treated}\;\text{LNP}}\times 100\%$$ (1)

### 2.5 Gene transfection of lipid nanoparticles

For transfection with pCAG-EGFP or pCas9-GFP, HEK293 cells were seeded in 12-well plates at a density of 1.0 × 10^5^ cells per well and incubated overnight to allow cell attachment. Lipid nanoparticles (LNPs) or Lipofectamine 3000 were then added to each well at a dose corresponding to 2 µg of pDNA per well and incubated for 24 h. After transfection, the cells were washed twice with DPBS and further incubated in fresh DMEM for an additional 48 h. After a total incubation period of 72 h, cells were detached using Trypsin–EDTA, resuspended in DPBS, and analyzed for GFP fluorescence using a Beckman Coulter Cytomics FC500 MPL flow cytometer (California, United States).

For transfection with pGuide-it plasmid, HEK293 cells were seeded in 12-well plates at a density of 3.0 × 10^5^ cells per well and incubated overnight, on the other hand THP-1 monocytes were seeded in 12-well plates at a density of 3.0 × 10^5^ cells per well before adding LNPs or Lipofectmine 3000. LNPs or Lipofectamine 3000 were then added to achieve a final pDNA dose of 2 µg per well, followed by incubation for 48 h. After transfection, cells were detached using Trypsin–EDTA and divided into two aliquots. One aliquot was resuspended in DPBS containing 5% 7-AAD solution for simultaneous analysis of ZsGreen expression and cell viability by flow cytometry using a BD FACSAria Fusion (New Jersey, United States). The other aliquot was collected and used for subsequent gene editing analysis.

### 2.6 Gene editing of lipid nanoparticles

HEK293 cells harvested as described in Section 2.5 were subjected to genomic DNA preparation for gene editing analysis. Cells were lysed using 100 µL of lysis buffer supplemented with 1 µL of proteinase K and incubated in an Applied Biosystems Veriti™ 96-well thermal cycler (Massachusetts, United States) under the following conditions: 60 °C for 5 min, 98 °C for 2 min, and then held at 25 °C. Subsequently, 5 µL of the resulting cell lysate was used as the template for PCR amplification. The PCR reaction mixture consisted of 25 µL of PCR Master Mix, 2.5 µL each of 10 µM forward and reverse primers, and 15 µL of nuclease-free distilled water. PCR amplification was performed under the following cycling conditions: initial denaturation at 98 °C for 30 s; 35 cycles of 98 °C for 10 s, 65 °C for 30 s, and 72 °C for 30 s; followed by a final extension at 72 °C for 2 min and a hold at 4 °C. The amplified PCR products were purified using a QIAquick PCR Purification Kit according to the manufacturer’s instructions. Purified amplicons were then subjected to Sanger sequencing performed by Eurofins Genomics Japan (Tokyo, Japan). Gene editing efficiency was quantified using the Tracking of Indels by DEcomposition (TIDE) algorithm (Brinkman et al., 2014). TIDE analysis was conducted by comparing Sanger sequencing chromatograms obtained from untreated control cells and cells treated with LNPs or Lipofectamine 3000. This method enables quantitative determination of genome editing efficiency and identification of predominant insertion and deletion (indel) patterns within the targeted genomic region.

## 3 Results

### 3.1 Preparation and characterization of LNPs

Lipid nanoparticles (LNPs) were prepared via microfluidic mixing, and their physicochemical properties were comprehensively characterized in Table 2. Particle size, polydispersity index (PDI), and zeta potential were measured. Prior to the measurements, each LNP sample was diluted with HEPES buffer (pH 7.4) to ensure a suitable scattering intensity. As summarized in Table 2, all LNPs formulations showed good homogeneity from low PDI values (<0.4), indicating a monodisperse size distribution. Initially, LNPs containing different pDNA cargos (pCas9-GFP or pCAG-EGFP) and helper phospholipids (DOPC or DOPE) were prepared using K3C16 as the main cationic lipid. Regardless of pDNA size or phospholipid type, these LNPs displayed hydrodynamic diameters in the range of approximately 105–130 nm and moderately positive zeta potentials (17–21 mV).

**TABLE 2:** Characterizations of the lipid nanoparticles.

LNP sample	Size distribution [nm]	PdI	Zeta potential [mV]	Loading efficiency [%]
K3C16-DOPC LNP (pCas9)	130.2 ± 71.7	0.305	17.3 ± 5.0	69
K3C16-DOPE LNP (pCas9)	107.5 ± 51.1	0.227	18.5 ± 5.6	83
K3C16-DOPE LNP (pCAG)	125.2 ± 51.6	0.170	21.3 ± 6.9	87
K3C14 LNP	116.3 ± 49.5	0.181	21.5 ± 4.6	91
K3C16 LNP	111.3 ± 48.1	0.187	33.3 ± 10.2	94
DOTAP LNP	102.0 ± 47.7	0.219	33.8 ± 9.0	85
SM-102 LNP	134.7 ± 48.6	0.130	4.0 ± 5.1	95

Furthermore, formulations containing a fixed pDNA cargo (pGuide-it) were prepared using different cationic or ionizable lipids. Under these conditions, both particle size and surface charge varied depending on the chemical structure of the main lipid component. Among the tested formulations, DOTAP-based LNPs exhibited the smallest mean diameter (102 nm), while lysine-type cationic LNPs (K3C14 and K3C16) showed slightly larger sizes (111–116 nm). The ionizable SM-102 LNP displayed the largest mean diameter (∼135 nm). In terms of surface charge, cationic LNPs (K3C14, K3C16, and DOTAP) showed positive zeta potential (25–30 mV) at a physiological pH, whereas the ionizable SM-102 LNP showed a near-neutral zeta potential, consistent with its pH-dependent ionization behavior. pDNA loading efficiency was quantified using the PicoGreen fluorescence assay, and all formulations achieved high encapsulation efficiencies ranging from 83% to 95%, although the DOPC-containing LNP showed a slightly lower encapsulation efficiency (69%).

For stability assessments (Figure 2), the K3C14, K3C16, DOTAP, and SM-102 LNPs were evaluated. After 40 days of storage in 20 mM HEPES buffer (pH 7.4) at 4 ℃ , all formulations demonstrated minor particle size growth but consistently maintained an average diameter well below the 200 nm stability threshold. Specifically, K3C14 and K3C16 LNPs reached final sizes of 119.6 nm and 126.8nm, respectively, while the SM-102 control remained stable at 138.7 nm. In terms of polydispersity, all nanoassemblies maintained PdI values significantly below 0.4, indicating sustained monodispersity over the testing period.

**FIGURE 2:**
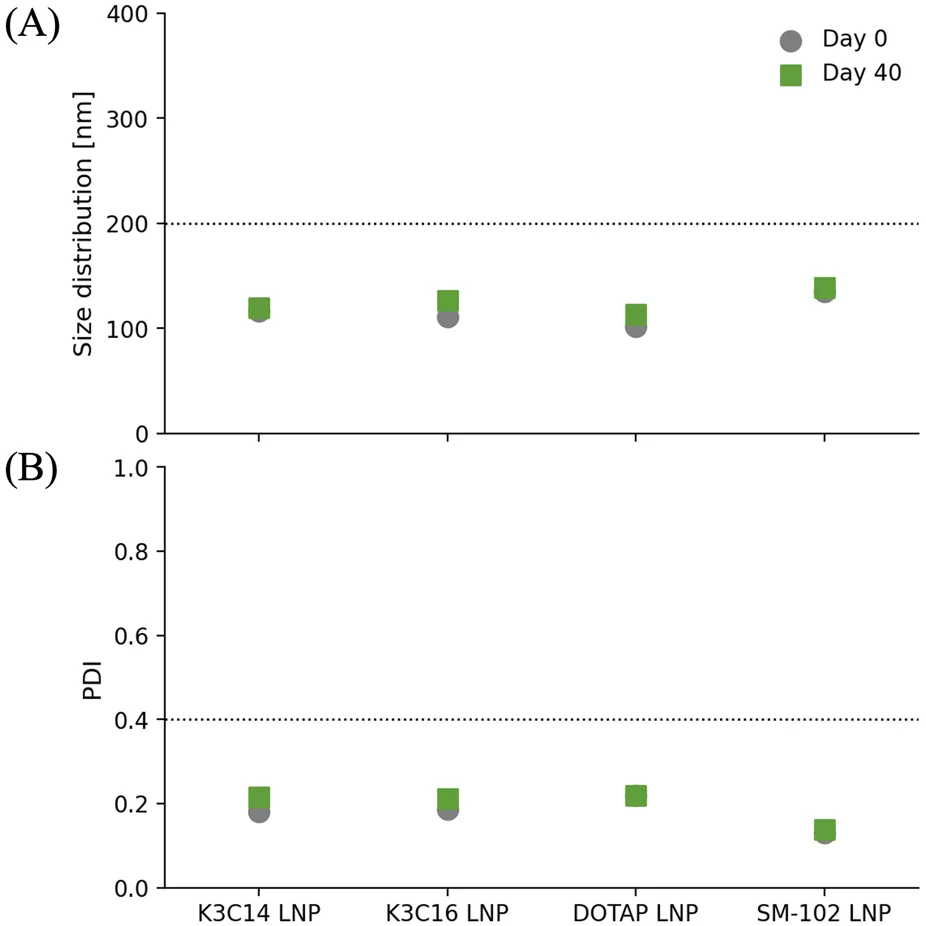
Colloidal stability assessments of LNP formulations over a 40-day storage period at 4 °C. **(A)** Long-term monitoring of hydrodynamic size distributions (nm) and **(B)** corresponding changes in the polydispersity index (PdI) for K3C14, K3C16, DOTAP, and SM-102 LNPs.

### 3.2 *In Vitro* pDNA transfection with different pDNA sizes and helper phospholipids

HEK293 cells were treated with LNPs or Lipofectamine 3000 for 24 h, followed by replacement with fresh medium and further incubation for 48 h. Transfection efficiency was evaluated by flow cytometry based on GFP fluorescence. As shown in Figure 3A, LNPs were prepared at a fixed lipid molar ratio (K3C16:phospholipid:cholesterol:PEG-DMG = 50:10:38.5:1.5), using either DOPC or DOPE as the phospholipid component, and loaded with the large plasmid pCas9-GFP. Under this formulation, DOPE-containing LNPs exhibited a significantly higher transfection efficiency (24.8%) compared with DOPC-containing LNPs (9.6%). This result suggests that DOPE is a more suitable helper phospholipid for K3C16-based LNPs when delivering large pDNA. After confirming DOPE as the preferred phospholipid, the effect of pDNA size on transfection efficiency was examined. As shown in Figure 3B, LNPs with identical lipid composition (K3C16:DOPE:cholesterol:PEG-DMG = 50:10:38.5:1.5) were loaded with either a smaller plasmid pCAG-EGFP (5521 bp) or a larger plasmid pCas9-GFP (9270 bp). LNPs carrying the smaller pCAG-EGFP plasmid achieved a markedly higher transfection efficiency (54.1%) than those carrying pCas9-GFP (29.6%). These results indicate that increasing pDNA size negatively affects transfection efficiency, even under optimized lipid composition.

**FIGURE 3:**
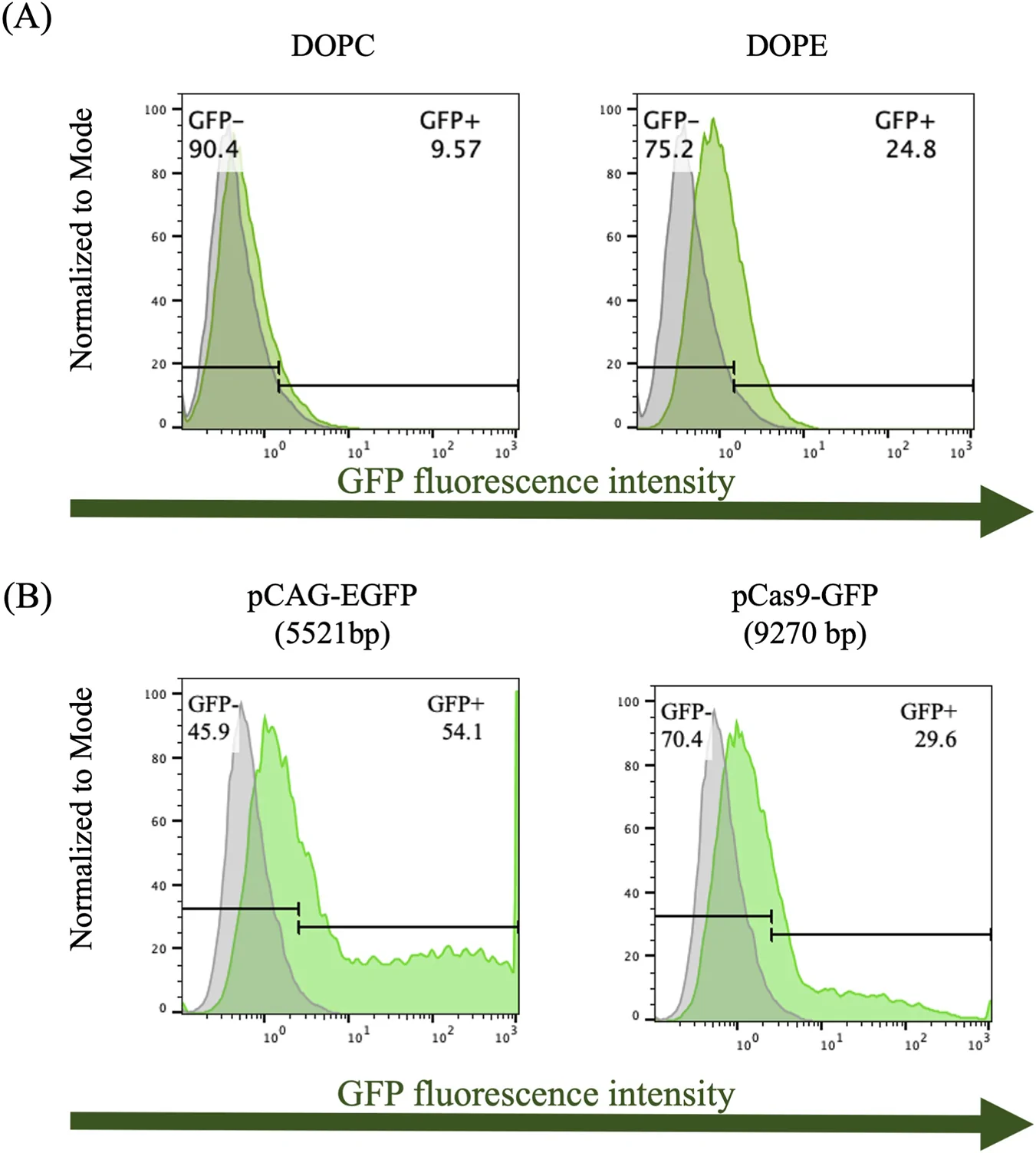
*In vitro* transfection performance of lysine-based LNPs. **(A)** Optimization of the helper lipid component using DOPE and DOPC formulations. **(B)** Expression profiles across different plasmid constructs and cargo lengths (pCAG-EGFP vs. pCas9-GFP).

### 3.3 *In Vitro* pDNA transfection and gene editing of LNPs

To assess the delivery versatility of the engineered lipid formulations across cell lineages, the LNP formulations and Lipofectamine 3000 were introduced to human THP-1 monocytes. In contrast to the results obtained in HEK293 cells, fluorescence microscopy revealed no detectable ZsGreen-positive cells across all tested formulations and biological replicates (Supplementary Figure S4).

HEK293 cells were treated with each LNP formulation or Lipofectamine 3000 for 48 h at a final concentration of 2 μg/mL pDNA encoding Cas9, sgRNA, and ZsGreen. Transfection efficiency was evaluated by flow cytometry based on ZsGreen fluorescence (Figure 4), expressed as the percentage of ZsGreen-positive cells and mean fluorescence intensity (MFI). As shown in the representative microscopy images (Figure 4A; Supplementary Figure S5A,C), cells treated with the various LNP formulations maintained high confluency. In contrast, bright-field observation of the Lipofectamine 3000-treated group revealed a marked reduction in cell confluency, suggesting potential impairment of cell proliferation. Corresponding fluorescence imaging demonstrated robust ZsGreen expression in cells treated with K3C14 LNP, K3C16 LNP, SM-102 LNP, and Lipofectamine 3000. Conversely, DOTAP LNP treated cells exhibited significantly lower levels of green fluorescence, a result consistent with the minimal transfection efficiency observed during flow cytometry analysis. As shown in Figures 4B,C, and Supplementary Figure S5B,D, both lysine-headgroup cationic formulations mediated robust gene transfer, with K3C14 LNP achieving an average transfection efficiency of 42.2% ± 5.3% and K3C16 LNP achieving 34.6% ± 5.8%, both markedly outperforming the conventional cationic benchmark DOTAP LNP (5.7% ± 3.8%). The ionizable SM-102 LNP formulation exhibited the highest overall transfection efficiency (90.2% ± 4.4%), followed by Lipofectamine 3000 (68.3% ± 11.6%). Consistent with these results, MFI analysis (Table 3) showed higher values for K3C14 LNP (29.3 ± 14.2) and K3C16 (24.2 ± 11.1) LNP compared to DOTAP LNP (2.3 ± 0.3), while remaining lower than SM-102 LNP (87.6 ± 47.2) and Lipofectamine 3000 (29.3 ± 14.2). These results indicate that K3C14 LNP and K3C16 LNP achieved substantially enhanced gene delivery performance compared with conventional cationic DOTAP LNPs.

**FIGURE 4:**
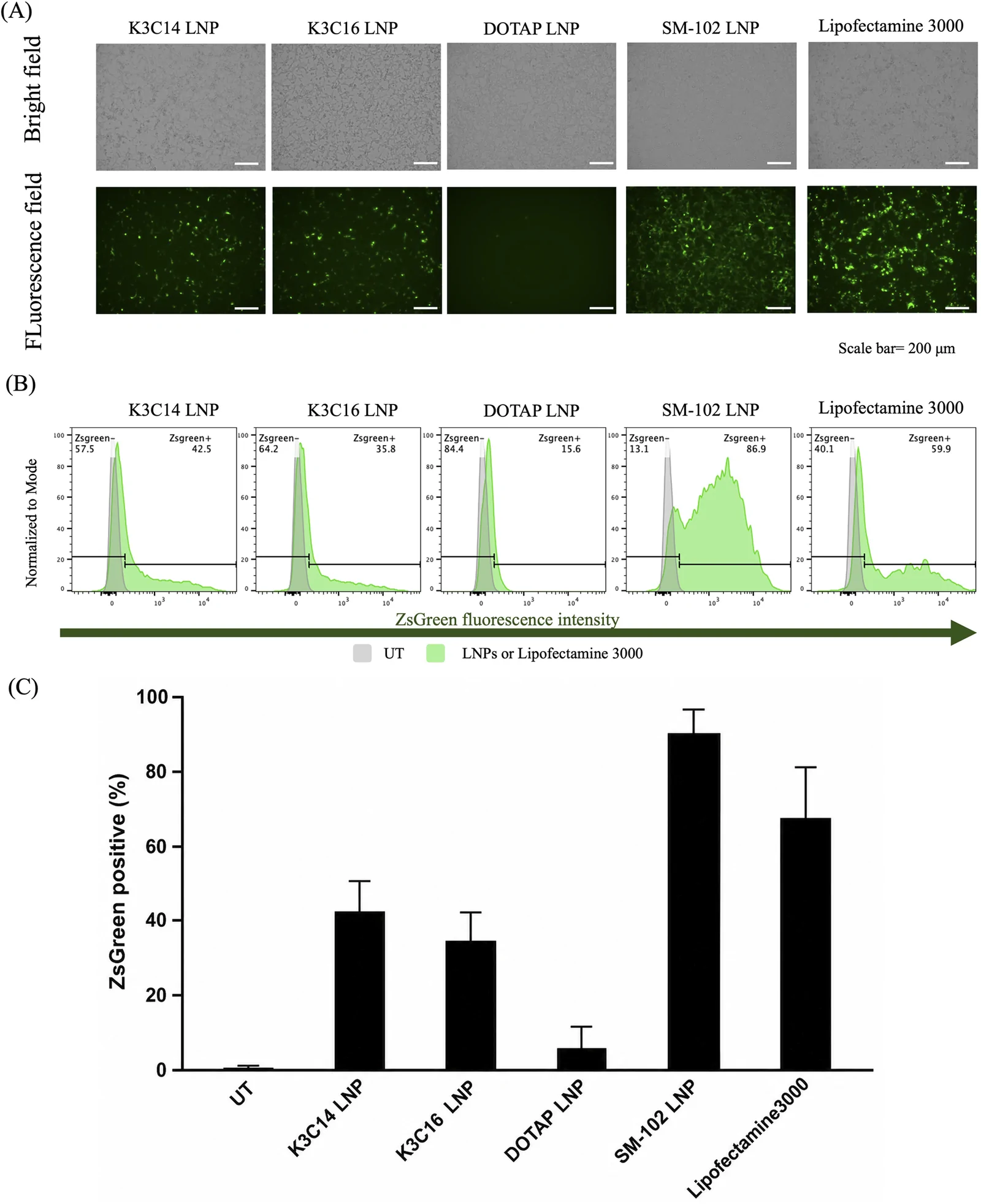
*In vitro* transfection efficiency and ZsGreen reporter expression in HEK293 cells after a 48 h treatment period. **(A)** Representative bright-field and fluorescence microscopy images evaluating cell confluency and intracellular ZsGreen distribution across various LNP formulations and Lipofectamine 3000. **(B)** Flow cytometry histograms quantifying the absolute percentage of ZsGreen-positive cell populations. **(C)** Mean percentage of ZsGreen positive cell populations, Data represent mean ± SD from 3 biological repeats.

**TABLE 3:** Normalized Mean Fluorescence Intensity (MFI) of HEK293 Cells.

Sample	MFI
K3C14	29.3 ± 14.2
K3C16	24.2 ± 11.1
DOTAP	2.3 ± 0.3
SM-102	87.6 ± 47.2
Lf3000	29.3 ± 14.2

UT is set to 1. Data represent mean ± SD from 3 biological repeats.

Cytotoxicity was analyzed by flow cytometry using 7-AAD staining (Figure 5; Supplementary Figure S6; Table 4) with cells localized in quadrants 1 and 2 defined as 7-AAD positive, representing the dead cell population. All LNP-treated groups exhibited low cytotoxicity (<3%). Total 7-AAD-positive cells accounted for 0.8% in DOTAP LNPs (0.8% ± 0.3% in Q1; 0.0% ± 0.0% in Q2), 0.7% in SM-102 LNPs (0.4% ± 0.2% in Q1; 0.3% ± 0.0% in Q2), 1.1% in K3C16 LNPs (0.9% ± 0.3% in Q1; 0.2% ± 0.1% in Q2), and 2.7% in K3C14 LNPs (1.9% ± 1.7% in Q1; 0.8% ± 1.0% in Q2). Lipofectamine 3000 showed a total 7-AAD-positive rate of 0.5% (0.2% ± 0.1% in Q1; 0.3 %± 0.1% in Q2) in flow cytometry.

**FIGURE 5:**
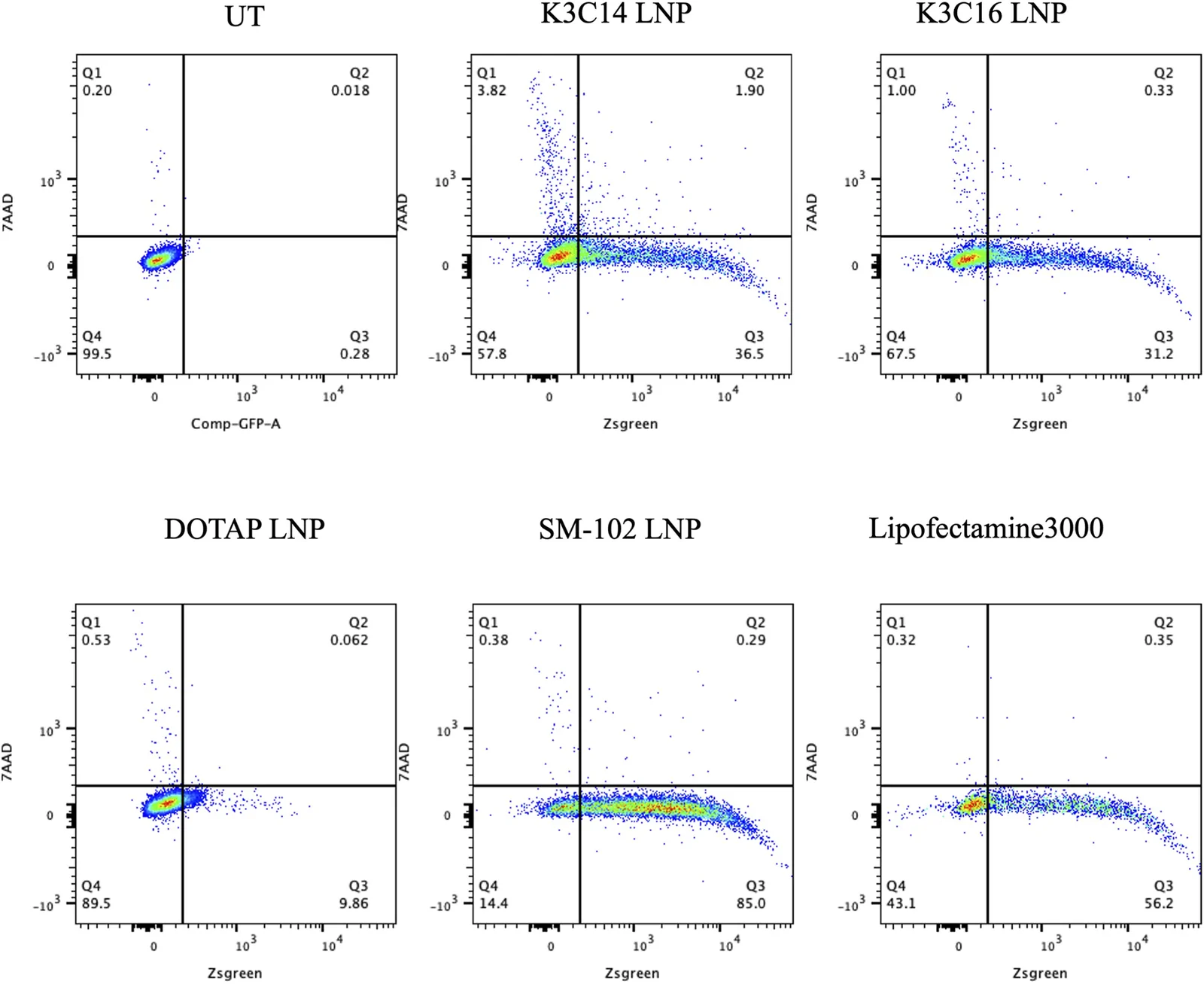
Flow cytometry dual-parameter dot plots evaluating LNP cytotoxicity. Two-dimensional dot plots demonstrate the simultaneous distribution of the ZsGreen transfection reporter (x-axis) and the 7-AAD cell death stain (y-axis) in HEK293 cells. Quadrant gating distinguishes the localization of dead cells (7-AAD positive) within both untransfected (ZsGreen-) and successfully transfected (ZsGreen+) cell populations.

**TABLE 4:** Quantitative evaluation of transfection efficiency and cytotoxicity in HEK293 cells via dual-parameter flow cytometry.

Sample	ZsGreen-/7AAD+ (%)	ZsGreen+ /7AAD+ (%)	ZsGreen-/7AAD- (%)	ZsGreen+ /7AAD- (%)
UT	0.2 ± 0.0	0.0 ± 0.0	99.6 ± 0.3	0.2 ± 0.2
K3C14	1.9 ± 1.7	0.8 ± 1.0	55.9 ± 4.8	41.4 ± 5.9
K3C16	0.9 ± 0.3	0.2 ± 0.1	64.5 ± 6.1	34.4 ± 5.8
DOTAP	0.8 ± 0.3	0.0 ± 0.0	93.4 ± 3.6	5.7 ± 3.7
SM-102	0.4 ± 0.2	0.3 ± 0.0	9.4 ± 4.4	89.9 ± 4.4
Lf3000	0.2 ± 0.1	0.3 ± 0.1	31.5 ± 11.5	68.1 ± 11.6

Data represent mean ± SD from 3 biological repeats.

Genome editing efficiency was evaluated using the TIDE algorithm, which compared Sanger sequencing data from LNP- or Lipofectamine 3000–treated cells to untreated controls. As summarized in Table 5, the editing efficiency trend mirrored that of transfection efficiency. Among the cationic LNPs, K3C14 LNP achieved the highest editing efficiency (14.5% ± 2.5%), followed closely by K3C16 LNP (13.4% ± 5.6%) and DOTAP LNP (7.6% ± 4.9%). The ionizable SM-102 LNP exhibited the highest overall editing efficiency (57.9% ± 9.9%), whereas Lipofectamine 3000 reached 27.4% ± 7.1%. Interestingly, although SM-102 LNP and Lipofectamine 3000 both showed comparably high transfection efficiencies (90.2% ± 4.4% and 68.3% ± 11.6%, respectively), their genome editing efficiencies differed by nearly twofold (57.9 ± 9.9%and 27.4% ± 7.1%, respectively). This observation suggests that while LNP mediated transfection effectively translated into Cas9 proteins and further formed Cas9-driven genome editing system, Lipofectamine 3000 mediated transfection did not convert into editing efficiency with the same proportionality.

**TABLE 5:** Gene editing efficiency of HEK293 cells.

Sample	Indel (%)
K3C14	14.5 ± 2.5
K3C16	13.4 ± 5.6
DOTAP	7.6 ± 4.9
SM-102	57.9 ± 9.9
Lf3000	27.4 ± 7.1

Data are shown as mean ± SD from 3 biological repeats.

## 4 Discussion

In this study, LNPs with different lipid compositions and pDNA cargos were prepared (Table 1) while maintaining comparable physicochemical properties, including particle size, polydispersity index (PDI), and surface charge. Among these formulations, the SM-102 LNP exhibited the largest particle size (134.7 nm), which is consistent with previous reports showing that ionizable LNPs formulated with DOPE as the phospholipid component tend to exhibit larger particle sizes than those containing DSPC (∼100 nm) (McMillan et al., 2024; Zhigaltsev and Cullis, 2023). Compared with ionizable SM-102 LNPs, cationic LNPs displayed slightly smaller particle sizes. This difference may arise from stronger electrostatic interactions between permanently charged cationic lipids and negatively charged pDNA during preservation at a physiological pH (pH7.4). While SM-102 LNPs are reported to form highly organized solid-core structures with small cargos like Antisense oligonucleotides (ASOs) or mRNA (He et al., 2026; Deng et al., 2025), the delivery of large, supercoiled pDNA (8327 bp) introduces significant structural constraints. The larger hydrodynamic diameter observed for the SM-102 LNPs formulations (134.7 nm) likely reflects the physical expansion required to accommodate the rigid plasmid scaffold as the ionizable lipids transition to a neutral state. In terms of surface charge, the SM-102 LNP exhibited a near-neutral zeta potential at a physiological pH (pH7.4), consistent with its lower pKa value and pH-dependent ionization behavior (He et al., 2025). In contrast, cationic LNPs (K3C14, K3C16, and DOTAP) showed positive zeta potentials in the range of 17–34 mV, reflecting their permanently charged headgroups. All formulations achieved pDNA encapsulation efficiencies of 70%–95%, indicating that a 50 mol% content of cationic or ionizable lipid enables efficient electrostatic complexation with pDNA during microfluidic mixing.

The first FDA-approved siRNA-LNP therapeutic, Patisiran (Adams et al., 2018), established a benchmark lipid molar ratio of ionizable lipid:DSPC:cholesterol:PEG-lipid = 50:10:38.5:1.5 (Hald et al., 2022). In the present study, this formulation strategy was adopted, with DSPC replaced by unsaturated phospholipids to better complement the lysine-based cationic lipid K3C16, which has been reported to exhibit high membrane fusogenicity (He et al., 2019; Hotta et al., 2023). To optimize the helper lipid component, LNPs containing either DOPC or DOPE were initially evaluated while keeping all other formulation parameters constant. The results demonstrated that DOPE-containing LNPs achieved significantly higher transfection efficiency than their DOPC-containing counterparts, leading to the selection of DOPE for subsequent formulations. By maintaining a benchmark molar ratio (50:10:38.5:1.5), we isolated the effect of phospholipid headgroup geometry. The conical architecture and H_II_ phase propensity of DOPE proved markedly superior to cylindrical DOPC, facilitating critical endosomal escape and vector unpacking necessary for functional genome editing. DOPE has been widely reported to enhance membrane fusion owing to its cone-shaped structure and ability to facilitate non-bilayer phase formation (Hammond et al., 2025; Martínez-Negro et al., 2019; Lonez et al., 2010; Álvarez-Benedicto et al., 2022), and its combination with K3C16 was therefore expected to further promotes membrane fusion–mediated cellular uptake or endosomal escape. Furthermore, the effect of pDNA size on transfection efficiency was investigated using plasmids of different lengths. LNPs carrying the larger Cas9-encoding plasmid (9270 bp) exhibited lower transfection efficiency than those carrying smaller pDNA (5521 bp), indicating that increasing plasmid size negatively impacts transfection performance. This observation is consistent with previous reports showing that large plasmids generally display reduced diffusional mobility and intracellular trafficking efficiency, thereby limiting effective gene delivery (Lukacs et al., 2000). To further clarify the biophysical parameters governing payload delivery, the sequence maps for pCAG-EGFP (5521 bp) and pCas9_GFP (9270 bp) have been provided in the Supplementary Material (Supplementary Figures S1, S2). As shown in the sequence architectures, both vectors utilize the identical upstream CAG promoter framework—comprising the CMV enhancer, chicken β -actin promoter, and a chimeric intron—to drive reporter expression. This structural commonality effectively eliminates variations in baseline promoter strength as a confounding factor. However, we acknowledge that the final green fluorescence readout cannot be attributed solely to physical plasmid size. While both constructs share the EGFP reporter sequence, pCAG-EGFP expresses standalone EGFP, whereas pCas9_GFP yields a massive, multi-domain Cas9-EGFP fusion protein. The distinct translation rates, complex protein folding kinetics, and potential variations in per-molecule fluorescence intensity of this large fusion construct act as unavoidable biological variables alongside the physical constraints of plasmid size. Nevertheless, the substantial increase in supercoiled hydrodynamic volume from 5521 bp to 9270 bp remains a primary biophysical impediment that restricts lateral cytoplasmic diffusion and passive nuclear translocation.

Building on the formulation screening described above, the transfection efficiencies of the LNPs and Lipofectamine 3000 carrying pGuide-it (8327 bp, encoding Cas9, guide RNA, and ZsGreen) were evaluated after 48 h of treatment on HEK293 cells. K3C14 LNP and K3C16 LNP exhibited markedly higher transfection and gene-editing efficiency compared with the commercial cationic LNP (DOTAP), while maintaining excellent biocompatibility. Although DOTAP has been reported to facilitate pDNA delivery (Cui et al., 2022), in this study, DOTAP-LNP showed the lowest transfection and editing efficiency. The quaternary ammonium headgroup of DOTAP provides a rigid, permanently positive charge without buffering capacity, resulting in strong electrostatic binding with pDNA but limited flexibility for cargo release. This rigidity likely impairs endosomal escape and hinders efficient transfection, especially for large nucleic acid cargos. In contrast, the lysine-derived headgroup of K3C14 and K3C16 contains a primary amine that would impart mild buffering ability and facilitate the disassembly of pDNA from LNP after cellular uptake. Previously, molecular assemblies of single lipid component were shown to engage distinct cellular entry routes driven strictly by alkyl tail length, with K3C14 internalized predominantly via classical endocytosis and K3C16 exhibiting pronounced direct plasma membrane fusogenicity (He et al., 2019). However, when translated into multi-component LNP with phospholipids, cholesterol, and PEG-lipids, the internal architecture shifts from a bilayer-based membrane structure to a densely packed core structure. Consequently, the direct membrane fusogenicity observed in bilayer structures may be attenuated by the compact lipids core. Even if K3C16 LNP are internalized through endocytosis, their fusogenic nature may promote interaction with endosomal membranes, facilitating endosomal escape and cytosolic release. Nevertheless, the overall transfection efficiency of K3C16 LNP remained lower than that of SM-102 LNP, indicating room for optimization in lipid composition. The current formulation and molar ratios were adapted from ionizable LNP optimized for mRNA delivery. To further enhance fusogenicity, the cholesterol ratio could be reduced to decrease membrane rigidity (Krause and Regen, 2014), the proportion of DOPE could be increased to promote nonbilayer structure formation (Lonez et al., 2010), and the PEG2000-DMG content could be lowered to expose more fusogenic lipid surface for interaction with cellular or endosomal membranes.

In contrast to their performance in HEK293 cells, none of the LNP formulations or Lipofectamine 3000 produced observable ZsGreen expression in THP-1 monocytes (Supplementary Figure S4). Monocytic suspension cells are well recognized as challenging targets for non-viral transfection, particularly for large supercoiled plasmid DNA payloads (>8 kb), owing to robust endo-lysosomal degradation pathways and limited nuclear entry. Future optimization efforts will focus on refining the lipid matrix composition—drawing on recent strategies tailored to overcome immune cell delivery barriers (Kulkarni et al., 2018; Lindsay et al., 2025) such as adjusting helper lipid ratios.

Flow cytometry analysis (Figure 5; Supplementary Figure S6; Table 4) demonstrated that all LNP formulations exhibited low overall cytotoxicity. Among them, notably, evaluation of the dead, transfected population (ZsGreen+/7-AAD+) revealed that K3C16 LNPs yielded a lower proportion of dead transfected cells (0.2% ± 0.1%) compared to SM-102 LNPs (0.3% ± 0.0%). These results suggest that the physicochemical structure and cellular uptake mechanism of SM-102 LNPs may induce greater cellular stress at high uptake levels than the lysine-based K3C16 LNPs. The ionizable lipid SM-102 increases its surface charge within the acidic endosomal environment to promote endosomal escape; however, incomplete escape of the cargo can lead to lysosomal rupture and subsequent cell death. This mechanism implies that higher uptake levels increase the likelihood of lysosomal disruption, resulting in a correlation between high transfection efficiency and elevated cytotoxicity (Bates et al., 2025). Lipofectamine 3000, which mediates transfection through strong cationic interactions and surfactant-like membrane disruption, similarly exhibited cell death within highly transfected populations despite high delivery efficiency. In contrast, the lysine headgroup of K3C16 LNPs confers superior biocompatibility, particularly in highly transfected cells. K3C16 has been reported to display low cytotoxicity (He et al., 2019). Consequently, K3C16 LNPs appear to be promising for prolonged, low-toxicity nucleic acid delivery, especially for plasmid DNA-based CRISPR/Cas9 systems, where sustained gene expression is advantageous compared with transient mRNA delivery (Hughes et al., 2009). This ability of K3C16 LNP to sustain delivery with minimal cytotoxicity is particularly valuable for repeated dosing or *ex vivo* applications such as gene-edited cell therapies. It is important to note a limitation inherent to our cytotoxicity profiling method. While 7-AAD staining provides a precise, quantitative measurement of acute cell death characterized by cell membrane permeabilization, it does not capture proliferation-based toxicity or cell-cycle arrest. This explains the observed divergence in the Lipofectamine 3000 group, which demonstrated a low acute 7-AAD signal during flow cytometry but displayed a marked reduction in cell confluency under bright-field microscopy. Future evaluations will incorporate metabolic or proliferation-tracking assays to comprehensively map long-term cytostatic effects alongside acute membrane damage.

The gene-editing efficiency was quantified using the TIDE algorithm based on Sanger sequencing data from LNP or Lipofectamine treated HEK293 cells compared with untreated controls. Both SM-102 LNP and Lipofectamine 3000 achieved high transfection efficiencies (90.2% ± 4.4% and 68.3% ± 11.6%, respectively). However, the proportion of transfected cells that underwent successful genome editing differed substantially between these systems. SM-102 LNP treated cells reached an editing efficiency of 57.9% ± 9.9%, whereas Lipofectamine 3000 treated cells achieved only 27.4% ± 7.1%. This difference suggests that although both systems effectively deliver plasmid DNA into cells, the intracellular expression kinetics and cell viability determine whether transfection events translate into functional CRISPR/Cas9 editing proteins. Bright-field microscopy (Figure 4A) revealed that Lipofectamine 3000 treated cells exhibited reduced confluency and impaired proliferation, implying that its surfactant-like mechanism induces significant membrane stress. Such stress would trigger transient overexpression of Cas9 and sgRNA, followed by rapid loss of cell viability before effective DNA cleavage and repair can occur (Shi et al., 2018; Rao et al., 2015). In contrast, SM-102 LNP-mediated delivery yielded both high transfection and superior editing efficiency, suggesting that the LNP formulation enables more stable and sustained gene expression. This may be attributed to its ionizable lipid chemistry, which balances efficient endosomal escape with relatively low long-term cytotoxicity. For K3C14 and K3C16 LNPs, the moderate transfection efficiency (42.2% ± 5.3% and 34.6% ± 5.8%, respectively) corresponded to a gene-editing rate of 14.5% ± 2.5% and 13.4% ± 5.6%, respectively, which are currently lower than the optimized SM-102 system, K3C14 and K3C16 demonstrated a functional baseline for all-in-one plasmid delivery without the severe membrane stress and impaired proliferation observed with Lipofectamine 3000. Given its low cytotoxicity and membrane-fusion capability, optimization of lipid composition and molar ratio may further improve nuclear delivery and editing outcomes. Overall, these findings emphasize that efficient genome editing requires not only high transfection rates but also biocompatible and kinetically favorable intracellular processing to sustain Cas9–sgRNA activity.

Despite the widespread success of LNPs for mRNA delivery, the use of LNPs for delivering large plasmid DNA (pDNA) encoding CRISPR/Cas9 remains significantly underreported in literature. Most non-viral CRISPR delivery strategies have pivoted toward mRNA or ribonucleoprotein (RNP) complexes because pDNA faces two major biological hurdles: the “stiffness” and large hydrodynamic size of supercoiled plasmids, which complicate efficient encapsulation, and the mandatory requirement for nuclear translocation to initiate transcription (Lukacs et al., 2000). While ionizable LNPs are highly effective for mRNA, their efficiency often drops precipitously when loaded with large pDNA (>8 kb) due to the increased complexity of cytosolic release and nuclear entry (Yin et al., 2017b). In this study, we successfully demonstrated that both lysine-based cationic LNPs and ionizable SM-102 LNPs could effectively package and deliver an 8.3 kb “all-in-one” CRISPR/Cas9 plasmid. The fact that K3C14 LNP and K3C16 LNPs achieved measurable gene editing (14.5% ± 2.5% and 13.4% ± 5.6%, respectively) with such a large cargo—while maintaining superior cell health—highlights its potential as a robust alternative to mRNA-based systems, which, despite higher transient efficiency, lack the long-term stability and manufacturing simplicity of plasmid-based platforms (Zu and Gao, 2021).

## 5 Conclusion

In this study, lysine-headgroup cationic LNPs were successfully developed to deliver a large, “all-in-one” CRISPR/Cas9 plasmid DNA. While nonviral delivery often prioritizes mRNA, our results demonstrate that pDNA remains a robust alternative for genome editing. By using an 8.3 kb construct encoding Cas9, sgRNA, and a reporter, we ensured stoichiometric consistency and functional editing *in vitro*. A critical finding was the superior biocompatibility of the K3C16-LNP platform. Although SM-102 and Lipofectamine 3000 achieved higher raw efficiency, they induced significant stress and death in highly transfected populations. In contrast, K3C16 LNPs maintained high confluency and viability, suggesting that the lysine-based headgroup and potential fusogenic pathway provide a gentler intracellular delivery mechanism. Despite the physical challenges of delivering large, supercoiled DNA across the nuclear barrier, K3C14 LNPs and K3C16 LNPs achieved measurable editing while preserving long-term cell health. These findings highlight lysine-based LNPs as a safe, cost-effective, and stable platform for CRISPR/Cas9 applications. Future optimization of lipid composition will further close the efficiency gap with ionizable systems, advancing the development of biocompatible vectors for sustained genome-editing therapies.

## Data Availability

The datasets presented in this study can be found in online repositories. The names of the repository/repositories and accession number(s) can be found in the article/Supplementary Material.
