# Supplementary material for: Overcoming delivery barriers of large all-in-one CRISPR/Cas9 plasmids using lysine-based lipid nanoparticles
Source: Front Genome Ed. 2026 Sep 16;8:1888079. doi: 10.3389/fgeed.2026.1888079 (PMC13624556; doi:10.3389/fgeed.2026.1888079)
Supplement: Supplementary file 1 [file Supplementaryfile1.docx]

Supplementary Material

# 1 Supplementary Figures and Tables

## 1.1 Supplementary Figures

**Supplementary Figure 1.** **Sequence map of pCAG-EGFP** (pCAG-EGFP was a gift from Wilson Wong (Addgene plasmid # 89684 ; http://n2t.net/addgene:89684 ; RRID:Addgene_89684))

**Supplementary Figure 2. Sequence map of pCas9-GFP** (pCas9_GFP was a gift from Kiran Musunuru (Addgene plasmid # 44719 ; http://n2t.net/addgene:44719 ; RRID:Addgene_44719)

**Supplementary Figure 3. Sequence map of pGuide-it (Green)** (Cat. No. 632601, Takara Bio/Clontech Laboratories)

**Supplementary Figure 4. ZsGreen reporter expression in THP-1 monocytes across independent biological replicates (A)Biological Replicate 1 (B)Biological Replicate 2 (C)Biological Replicate 3**

**Supplementary Figure 5. *In vitro* transfection efficiency and ZsGreen reporter expression in HEK293 cells across independent biological replicates (Replicates 2 and 3).**

(**A, B**) **Biological Replicate 2 (C, D**) **Biological Replicate 3**

**Supplementary Figure 6. Dual-parameter flow cytometry cytotoxicity profiling in HEK293 cells across independent biological replicates (Replicates 2 and 3).**

Two-dimensional flow cytometry dot plots display the simultaneous distribution of ZsGreen reporter expression (x-axis) and 7-AAD cell death stain (y-axis) 48 h post-transfection across untreated control (UT), lysine-based LNPs (K3C14, K3C16), commercial LNP (DOTAP, SM-102), and Lipofectamine 3000.

(**A**) **Biological Replicate 2** (**B**) **Biological Replicate 3**
